# Predicting the potential distribution of *Corylus heterophylla* in China under future climate change using an optimized MaxEnt model

**DOI:** 10.3389/fpls.2025.1668828

**Published:** 2025-11-17

**Authors:** Jingyu Wang, Wang Xing, Pengfei Sun, Dali Liu, Chunxiang Cheng

**Affiliations:** 1 National Beet Medium-Term Gene Bank, Heilongjiang University, Harbin, China; 2 Key Laboratory of Beet Genetics and Breeding, College of Modern Agriculture and Ecological Environment, Heilongjiang University, Harbin, China; 3 China National Seed Group Co., Ltd., Sanya, China; 4 Heilongjiang Climate Center, Harbin, China; 5 Heilongjiang Ecological Meteorological Center, Harbin, China

**Keywords:** *Corylus heterophylla*, suitable area, climate change, MAXENT model, CMIP6

## Abstract

**Introduction:**

*Corylus heterophylla* is native to East Asia, including northern and central China, southeastern Siberia, eastern Mongolia, Korea, Japan, and other adjacent regions, and its geographical distribution is highly sensitive to climate change. Investigating shifts in its suitable habitat under 1970-2000 and future climate conditions is crucial for the conservation and sustainable utilization of its germplasm resources.

**Methods:**

This study employed an optimized MaxEnt model, integrating species occurrence records with multiple environmental variables, to simulate and analyze potential suitable habitats and their key environmental determinants under various climate scenarios.

**Results:**

The results demonstrated that the model configured with the feature combination (FC) of LQPH and a regularization multiplier (RM) of 3 achieved low complexity, minimal overfitting, and high predictive accuracy (AUC = 0.933). The dominant factors influencing the distribution were identified as Bio16 (Precipitation of wettest quarter, 39.5%), Bio9 (Mean temperature of driest quarter, 22.2%), Alt (Altitude, 16.2%), and Bio3 (Isothermality, 7.1%). The 1970-2000 climatically suitable area for *C. heterophylla* spans approximately 210.85 × 10^4^ km^2^, accounting for 21.96% of China’s total land area. Projections under future climate scenarios indicate that the suitable habitat area for *C. heterophylla* will decrease slightly, primarily in low suitability zones, while high and medium suitability zones will expand. Its distribution pattern is expected to shift significantly northward while contracting southward, with the distribution centroid moving toward higher latitudes.

**Discussion:**

These findings provide a scientific basis for conserving and sustainably utilizing *C. heterophylla* under climate change.

## 1 Introduction

Global climate change significantly impacts biodiversity and potential ecological responses, making this a major research focus (Trew and Maclean, 2021). These responses encompass a range of phenomena, including shifts in species distribution, alterations in phenology, and the restructuring of ecological communities, which ultimately threaten ecosystem stability (Rubenstein et al., 2023). Climate-driven alterations in species geographical distributions exacerbate biodiversity and germplasm resource loss, and accelerate extinction rates (Bellard et al., 2012). The Intergovernmental Panel on Climate Change (IPCC) Sixth Assessment Report (AR6) highlights that ecological risks from global warming may cause irreversible impacts on ecosystems and human societies (Kuang et al., 2022). Many evidence indicates climate warming reduces species’ habitat ranges (Lenoir et al., 2020), frequently driving migration towards higher altitudes and latitudes (Chen et al., 2011). Consequently, assessing the potential distribution of species under climate change is critical for biodiversity conservation and climate adaptation. Predicting future changes in suitable habitats not only offers a scientific foundation for developing proactive biodiversity strategies and enhancing ecosystem resilience, but also provides theoretical support for the preservation of germplasm resources and the design of climate-informed ecological management practices.

*Corylus heterophylla* Fisch. ex Trautv. (*C. heterophylla*), an ecologically and economically significant species within *Corylus* (Betulaceae), dominates northeastern, northwestern, and northern China’s mountainous forests (Cheng et al., 2019). Its extensive root system mitigates soil erosion, contributing to vegetation restoration and ecosystem rehabilitation (Wang et al., 2025). Current research on *C. heterophylla* emphasizes phenotypic traits (Fan et al., 2020), genetic diversity (Yang et al., 2023), and cultivation techniques (He et al., 2023), with limited focus on its ecological niche requirements. Previous research on *Corylus* distribution has often examined *C.xheterophylla* as part of broader species groups. For instance, Liu and Chen (2024) modeled the potential distribution of 18 hazel species across China under current and future climate scenarios, integrating climatic, topographic, and anthropogenic variables. In a related effort, He et al. (2023) reconstructed the historical distribution changes of the *C. heterophylla* complex since the Last Interglacial. While these studies offer valuable macro-scale insights, the ecological distinctiveness of *C. heterophylla* may not be fully resolved in multi-species or complex-level analyses due to potential niche overlap among congeners. Species-specific distribution modeling helps minimize such interspecific interference and is essential for accurately capturing a target species’ unique climatic responses and spatial patterns. It also forms the basis for developing tailored conservation and sustainable management strategies. Therefore, this study focuses exclusively on *C. heterophylla* to clarify its niche characteristics and response mechanisms to critical environmental factors—addressing an important gap in fine-scale, single-species distribution modeling under future climate scenarios.

Species Distribution Models (SDMs) are widely used to study species–environment relationships and predict geographical distributions (Zurell et al., 2020). Common algorithms include CLIMEX (climate explorer) (Sutherst and Maywald, 1985), MaxEnt (Phillips et al., 2006), and BIOMAPPER (biogeographical mapping and modelling) (Hirzel and Guisan, 2002). These models apply statistical methods to correlate species occurrence with environmental conditions, generating habitat suitability indices based on niche theory (Zurell et al., 2020). Among them, MaxEnt has been widely applied in species distribution modeling due to its low data requirements, high predictive stability, and strong interpretability (Brown et al., 2017). The model’s primary strength lies in its ability to construct high-performance and robust species distribution models using presence-only data (Valavi et al., 2022). The model also supports quantitative evaluation of environmental variable contributions, allows rigorous assessment of variable importance through jackknife tests, and enables reliable validation of predictive performance using receiver operating characteristic (ROC) curves and the area under the curve (AUC) (Phillips et al., 2006). Furthermore, parameter optimization approaches (Chen et al., 2025; Fianchini et al., 2025) can enhance the model by reducing overfitting and improving generalizability and reliability (Guisan et al., 2013). With the capacity to incorporate future climate scenarios, MaxEnt can systematically assess potential climate change impacts on habitat suitability (Elith and Leathwick, 2009). Given these advantages, MaxEnt is well-suited for species with restricted ranges and complex niches, and was therefore selected as the modeling framework in this study.

In this study, we employed an optimized MaxEnt model with *C. heterophylla* occurrence data and environmental variables (climate, soil, topography) to predict current and future suitable habitats (Lee et al., 2021). Specific objectives include: (1) identify key environmental drivers governing *C. heterophylla* distribution and quantify their relative contributions; (2) classify habitat suitability levels and analyze spatial patterns of the species; (3) project future changes in suitable habitat area and range shifts under multiple climate scenarios (SSP1-2.6, SSP2-4.5, SSP5-8.5), elucidating the species’ distributional response mechanisms to climate change for evidence-based conservation strategies.

## 2 Materials and methods

### 2.1 Occurrence data collection and processing

**Figure 1:**
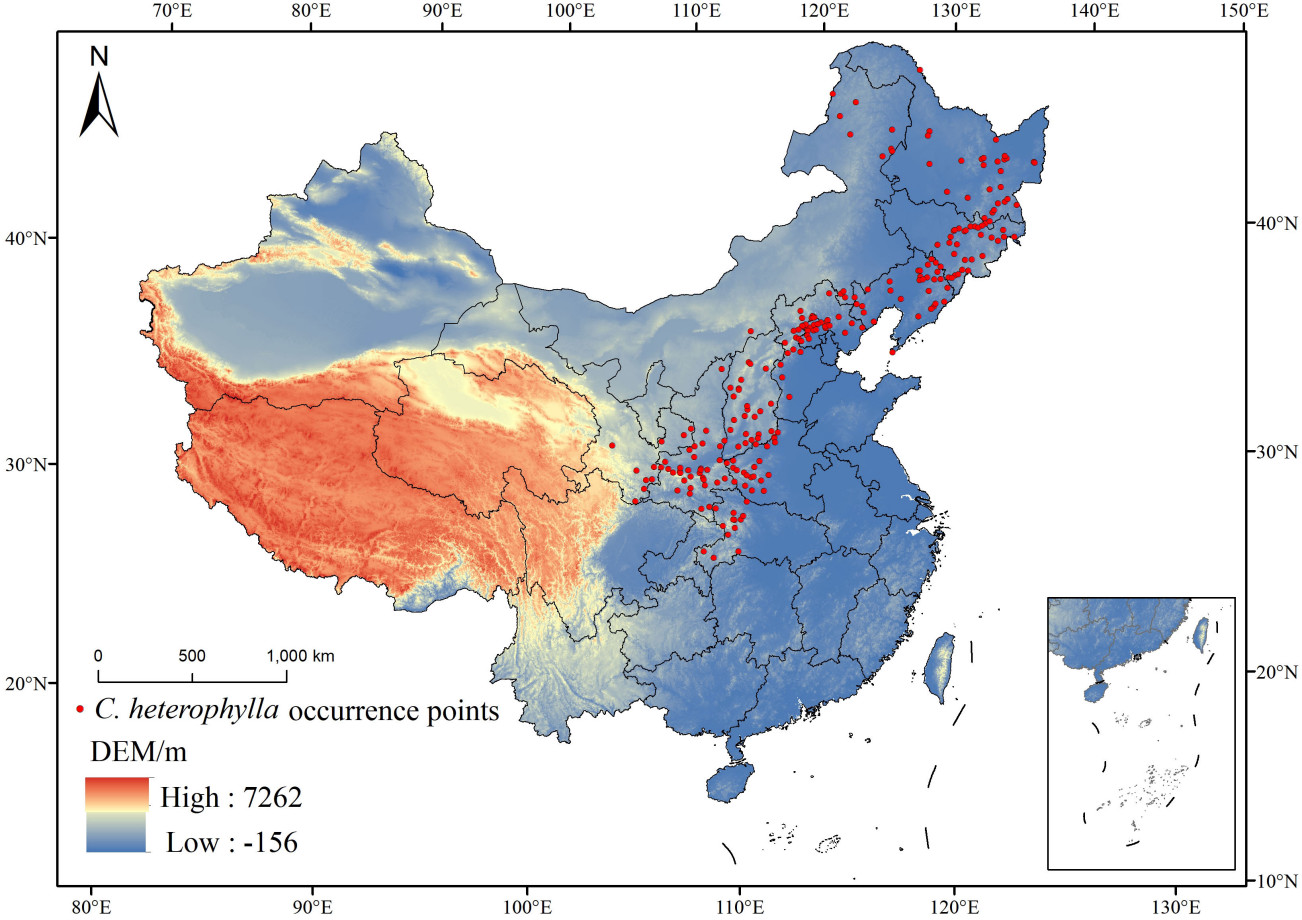
Occurrence points of *C. heterophylla* in China.

*C. heterophylla* occurrence records across China were compiled from authoritative sources: the Global Biodiversity Information Facility (GBIF; https://www.gbif.org/), Chinese Virtual Herbarium (CVH; https://www.cvh.ac.cn/), Plant Photo Bank of China (PPBC; https://ppbc.iplant.cn/), and published literature (He et al., 2023; Liu and Chen, 2024). Records lacking precise coordinates were georeferenced using Baidu Map Coordinate Picker (https://api.map.baidu.com/lbsapi/getpoint/index.html) based on textual descriptions, and corresponding uncertainties were estimated according to the specificity of each location description. To mitigate the effects of spatial autocorrelation and sampling bias inherent in herbarium and literature-based data (Taylor et al., 2020), the dataset was rigorously cleaned by removing duplicate and ambiguous records. Spatial thinning was then performed using the R package spThin (Allouche et al., 2006), with a filtering distance of 5 km to ensure that only one occurrence record was retained per 5 ×5 km grid cell (Wang et al., 2024). The final dataset comprised 243 spatially independent occurrence records (**Figure 1**), which were formatted into a CSV file for input into the MaxEnt model.

### 2.2 Environmental variable selection

Thirty-one environmental variables potentially influencing the distribution of *C. heterophylla* were initially selected, comprising 19 bioclimatic variables (under CMIP6 SSPs scenarios) (Urban et al., 2016), 9 soil parameters, and 3 topographic factors. In this study, soil parameters and topographic factors are generally considered to remain constant under future climate projections. To reveal its core climatic niche, the model excludes disturbance factors such as human activities, thereby simulating potential suitable distribution areas under future climate change (Ren et al., 2025; Shan et al., 2025). 1970–2000 climate data and future climate projections for the 2050s (2041-2060) and 2090s (2081-2100) were obtained from WorldClim (version 2.1; http://www.worldclim.org) at 2.5 arc-minute resolution. Future climate projections were derived from the BCC-CSM2-MR model developed by the China Meteorological Administration (CMA), considering three Shared Socioeconomic Pathways (SSPs): SSP1-2.6 (low radiative forcing), SSP2-4.5 (intermediate), and SSP5-8.5 (high) (Wang et al., 2024). Soil data came from the Harmonized World Soil Database (HWSD v1.2; Cold and Arid Regions Scientific Data Center), while topographic data were obtained from Geospatial Data Cloud (http://www.gscloud.cn). All topographic and soil datasets were resampled to a 2.5 arc-minute resolution using ArcGIS 10.8 to ensure consistency with the climatic data layers.

To mitigate model overfitting caused by multicollinearity among environmental variables (Wang et al., 2020), we performed a structured variable selection procedure. Initial MaxEnt runs were conducted to evaluate variable importance based on percent contribution and permutation importance. A variable was excluded only if both of these values were exactly 0% and its response curve in the jackknife test fully overlapped with the curve generated using all variables. Environmental factor correlations were analyzed using IBM SPSS Statistics (version 28.0; IBM Corp., Armonk, NY, USA). By combining the contribution rates of variables with correlation matrix analysis, factors exhibiting pairwise correlation with the coefficient |r| > 0.8 underwent selection refinement: the lower-contribution variable in each correlated pair (determined from preliminary analysis) was eliminated (Yi et al., 2016). Further consideration was given to thexbiological plausibility of each variable, evaluating its association with the ecological requirements of *C. heterophylla* and substantiating findings through ecological theory. For instance, the selected variables are closely linked to the species’ water requirements and temperature adaptability, exhibiting not only statistical significance but also clear biological plausibility. This process yielded nine key environmental factors for final model construction (**Table 1**).

**Table 1:** Environmental variables used in MaxEnt modeling.

Variable code	Environmental factor	Unit
Bio16	Precipitation of wettest quarter	mm
Bio9	Mean temperature of driest quarter	°C
Alt	Altitude	m
Bio4	Temperature seasonality (standard deviation ×100)	–
Bio19	Precipitation of coldest quarter	mm
Slope	Slope	%
REF_Depth	Reference depth	cm
Bio3	Isothermality (Bio2/Bio7×100)	–
T_bs	Base Saturation (Topsoil)	cm

### 2.3 Model construction

MaxEnt v3.4.4 was used to predict the suitable habitats for *C. heterophylla* across three time periods. The model incorporated 243 occurrence points with 25% randomly allocated for testing and 10 replicate runs. Outputs were formatted as logistic probabilities (0 to 1). Environmental variable importance was assessed through jackknife testing, with key factors identified by joint consideration of percent contribution and permutation importance. Model accuracy was evaluated using the Area under the Receiver Operating Characteristic (ROC) Curve (AUC) (Phillips et al., 2006). AUC values (0–1 scale) indicate predictive accuracy: <0.6 (failed), 0.6-0.7 (poor), 0.7-0.8 (fair), 0.8-0.9 (good), and 0.9-1.0 (excellent) (Zhang et al., 2024).

### 2.4 Model optimization

Default MaxEnt parameters may produce overly complex models prone to overfitting (Morales et al., 2017). In this study, the regularization multiplier (RM) and feature combination (FC) in the MaxEnt model were optimized using R’s Kuenm package (Cobos et al., 2019). From baseline settings (RM = 1, FC=LQHPT), RM was tested across 0.5-4.0 (0.5 increments) with randomized combinations of five feature classes: linear, quadratic, product, threshold, and hinge. *C. heterophylla* occurrence data were randomly partitioned (75% for training, 25% for testing). Model complexity was tested based on the evaluated using δAICc values and 5% missing rates, where lower δAICc indicates superior predictive performance (Giraldo and Kucuker, 2024).

### 2.5 Habitat suitability classification

MaxEnt output files (ASC format) were batch-converted to raster format in ArcGIS. Predicted suitability for *C. heterophylla* was reclassified into four categories based on mean logistic values: unsuitable (0-0.2), low suitability (0.2-0.4), medium suitability (0.4-0.6), and high suitability (0.6-1) (Gu et al., 2021; Xu et al., 2019). Distribution map for 1970-2000, 2050s and 2090s periods were generated using a China basemap. Suitable habitat areas were quantified per period using ArcGIS Spatial Analyst tools.

### 2.6 Analysis of spatial dynamics and centroid shifts

Based on the potential habitat distribution maps for 1970–2000 and future periods generated by the MaxEnt model, we quantitatively analyzed spatial pattern changes using the Distribution Change Analysis module in SDM toolbox (v2.0). Habitat transitions were classified into four categories (-1: range expansion; 0: species absence; 1: stable habitat; 2: range contraction). Spatial change maps were generated in ArcGIS 10.8. Centroid positions for 1970–2000 and future suitable habitats were calculated using the Centroid Changes (Liners) tool in SDM toolbox (v2.0), with migration distances indicating directional shifts in habitat distribution.

## 3 Results

### 3.1 Model performance and validation

**Figure 2:**
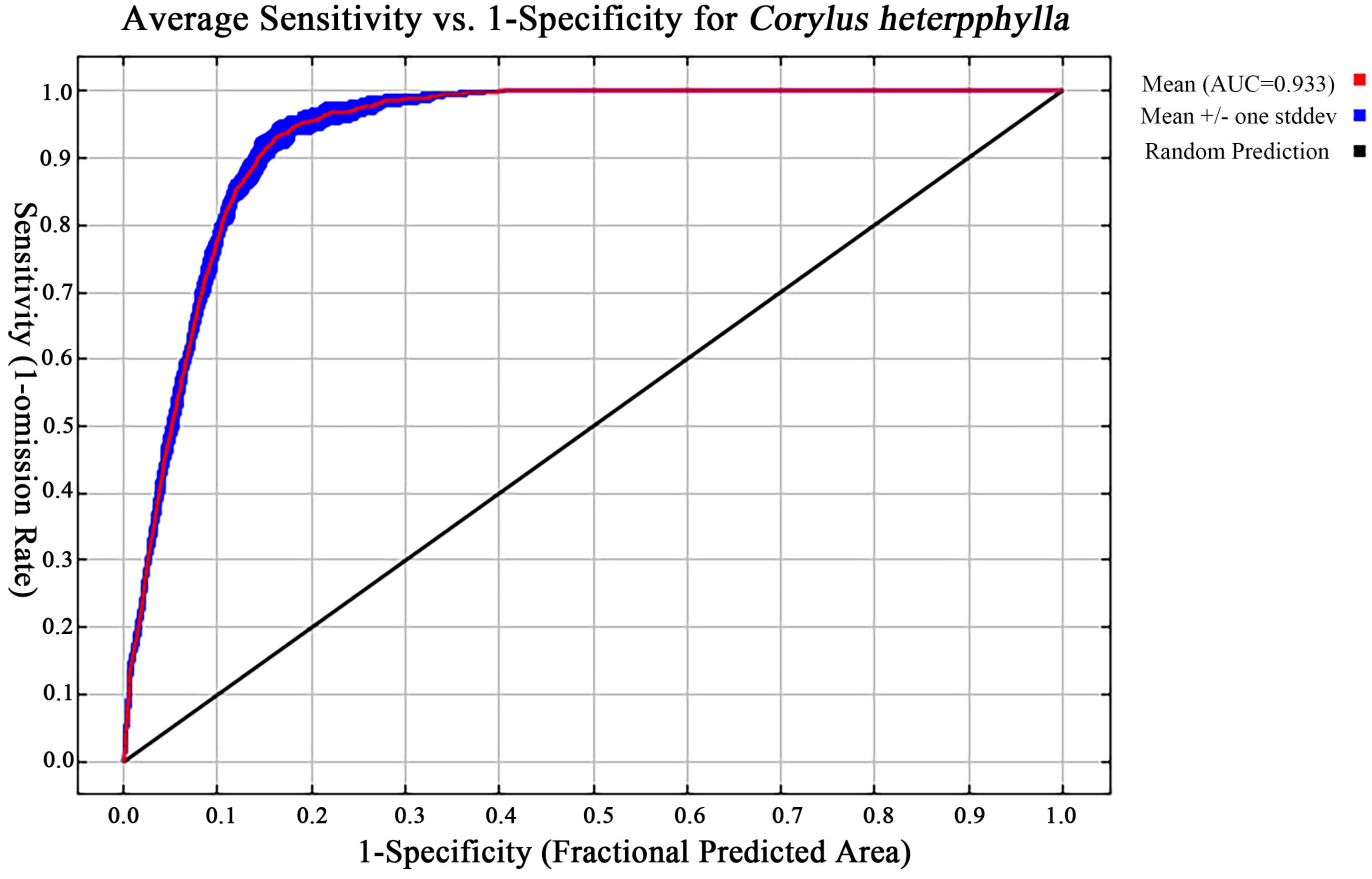
Receiver operating characteristic (ROC) curve for *C. heterophylla* habitat suitability modeling using MaxEnt.

The MaxEnt model simulated *C. heterophylla* potential habitats using 243 occurrence records and nine environmental factors. Default parameters (RM = 1, FC=LQPTH) yielded δAICc=88.48, whereas optimized parameters (RM = 3, FC=LQPH) achieved δAICc=0, indicating superior model performance (**Table 2**). Ten cross-validations with optimized settings produced a mean AUC of 0.933, confirming high predictive accuracy for habitat suitability (**Figure 2**).

**Table 2:** Performance evaluation of MaxEnt models under different parameter settings.

Model evaluation	Feature combination	Regularization multiplier	δAICc	5% training omission rate
Default	LQPTH	1	88.48	0.114754098
Optimized	LQPH	3	0	0.049180328

### 3.2 Environmental factors affecting the distribution of *C. heterophylla*

**Figure 3:**
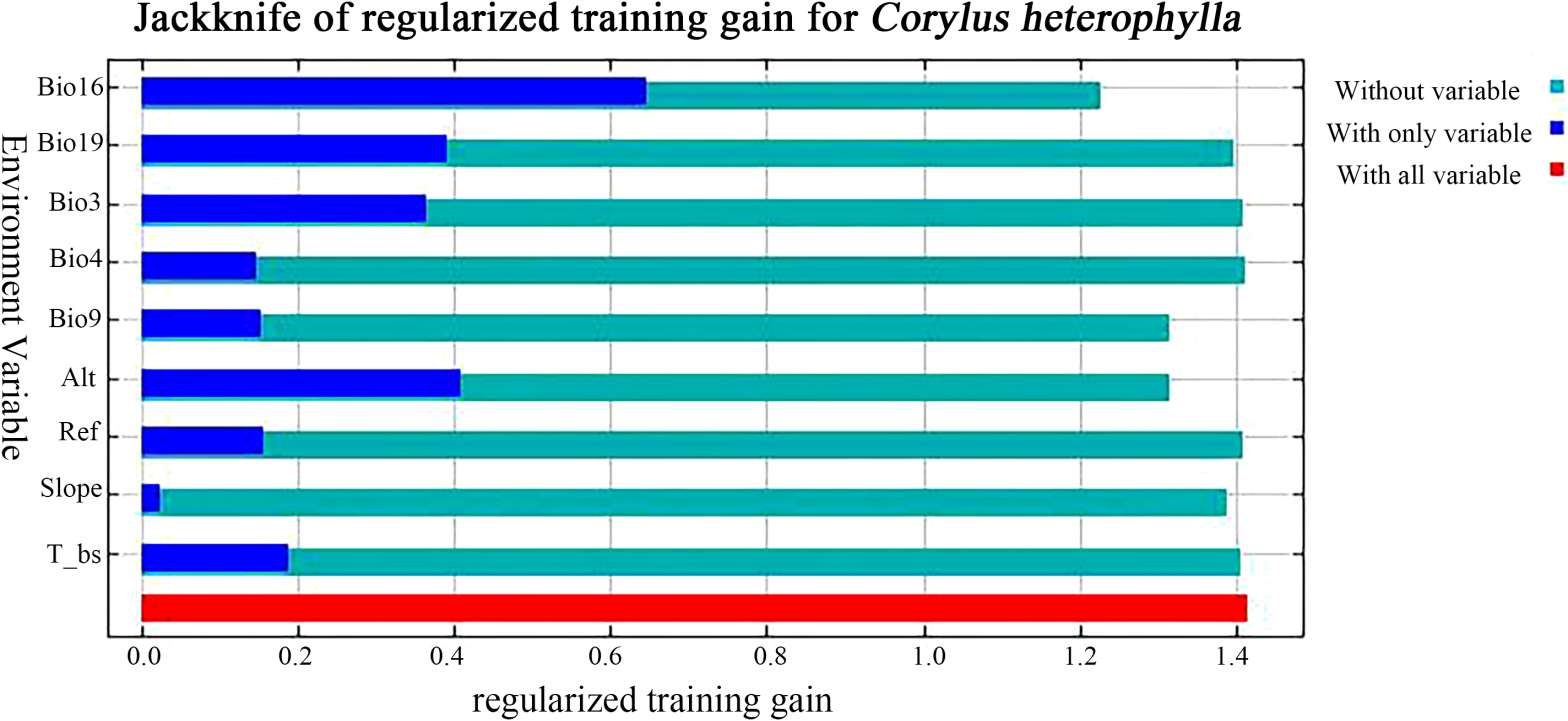
Jackknife test of regularized training gain for environmental variables.

Analysis of regularization training gain, permutation importance, and jackknife tests from the MaxEnt model simulation identified key environmental factors influencing *C. heterophylla* distribution. Bio16 (Precipitation of wettest quarter) showed highest contribution (39.5%), followed by Bio9 (Mean temperature of driest quarter, 22.2%), Alt (Altitude, 16.2%), and Bio3 (Isothermality, 7.1%), collectively accounting for 85% of total contribution. Permutation importance highlighted Bio16 (41.1%) and Alt (27.6%) as most significant, totaling 68.7% (**Table 3**; **Figure 3**). In contrast, the remaining variables—REF Depth (4.0%), Bio19 (3.9%), T_bs (3.5%), Slope (2.3%), and Bio4 (1.2%)made markedly lower contributions, collectively accounting for only 14.9%. Results indicate that precipitation during Bio16 (Precipitation of wettest quarter) constitutes the dominant factor in the model. Alt (Altitude) is identified as a secondary yet critical determinant, while temperature-related variables (Bio9, Bio3) further refine habitat suitability. Together, these form the core ecological drivers for *C. heterophylla.*

**Table 3:** Contribution rates and permutation importance of environmental factors governing *C. heterophylla* distribution.

Variable	Percent contribution (%)	Permutation importance (%)
Bio16	39.5	41.1
Bio9	22.2	20.9
Alt	16.2	27.6
Bio3	7.1	0.8
REF_Depth	4.0	1.4
Bio9	3.9	4.2
T_bs	3.5	0.5
Slope	2.3	2.3
Bio4	1.2	0.8

**Figure 4:**
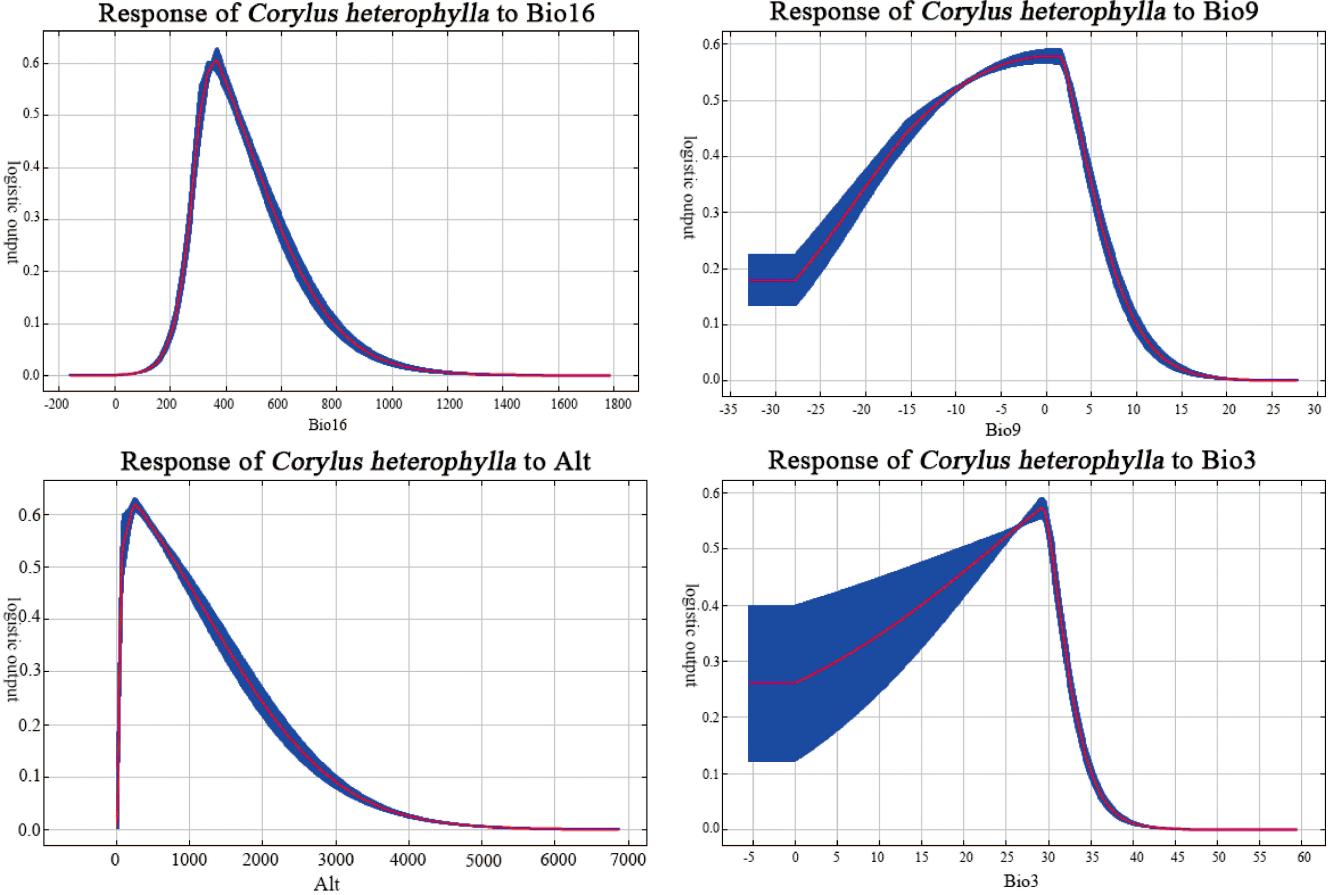
Response curves of *C. heterophylla* occurrence probability to key environmental factors.

*C. heterophylla* occurrence probability followed unimodal responses to environmental gradients (**Figure 4**). Using probability > 0.5 as threshold (Araújo et al., 2019) the optimal range for Bio16 (Precipitation of the Wettest Quarter) was found to be between 300 mm and 450 mm, with the highest probability (approximately probability = 0.6) occurring at 350 mm. The suitable range for Bio9 (The mean temperature of the driest quarter) was -12°C to 2°C, peaking at -2°C (approximately probability = 0.56). The optimal Alt (Altitude) range was 100 to 700 m, with a peak at 300 m (approximately probability = 0.63). For Bio3 (Isothermality), the suitable range was between 24 and 30, peaking at 28 (approximately probability = 0.57).

### 3.3 Projections of *C. heterophylla* distribution across temporal scales

#### 3.3.1 1970–2000 habitat suitability

**Figure 5:**
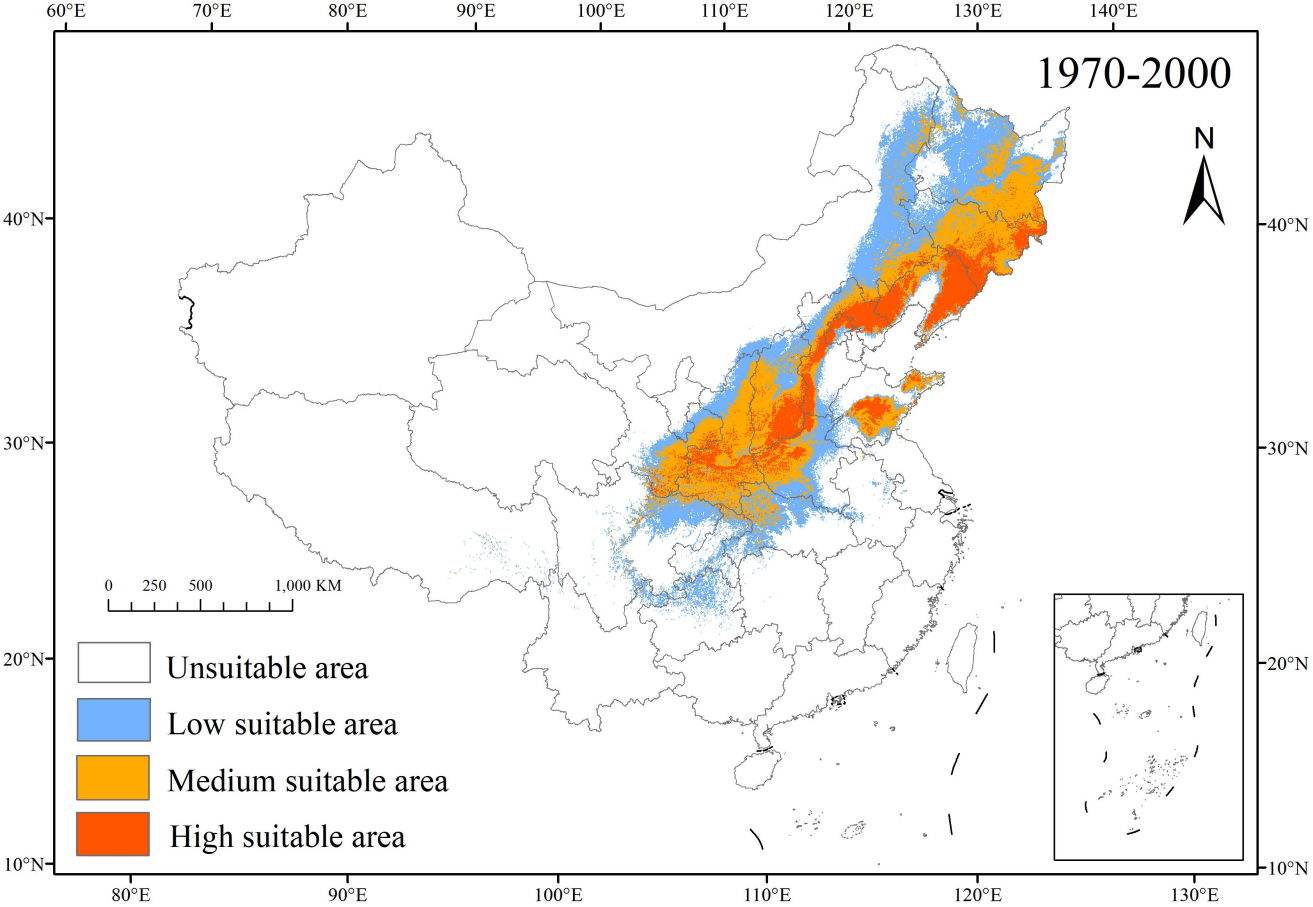
1970–2000 distribution of *C. heterophylla* in China.

Under 1970–2000 conditions (**Figure 5**), *C. heterophylla* primarily inhabits northeastern and northern China (Heilongjiang to Hubei; 21.96% of land area). Total suitable habitat spans 210.85 × 10^4^ km^2^, comprising low-suitability zones (108.37 × 10^4^ km^2^, 11.29%) concentrated in Henan, Sichuan, Chongqing; medium-suitability zones (63.39 × 10^4^ km^2^, 6.6%) predominantly in Heilongjiang, Inner Mongolia, Henan, and Gansu; and high-suitability zones (39.09 × 10^4^ km^2^, 4.07%) focused in Jilin, Liaoning, Hebei, Shanxi (**Table 4**). A comparison between the observed distribution points of *C. heterophylla* (**Figure 1**) and its predicted potential distribution (**Figure 5**) reveals that the actual occurrences predominantly reside within medium-high suitability areas.

**Table 4:** Potential suitable habitat area (×10^4^ km^2^) for *C. heterophylla* under 1970–2000 and future climate scenarios.

Suitable area types	1970-2000	2050s			2090s		
		SSP1-2.6	SSP2-4.5	SSP5-8.5	SSP1-2.6	SSP2-4.5	SSP5-8.5
Low suitable	108.37	67.99	64.14	61.42	69.82	60.31	44.71
Moderately suitable	63.39	83.18	89.59	88.13	80.49	87.93	96.29
Highly suitable	39.09	52.08	49.00	57.17	51.07	52.68	74.25
Total suitable	210.85	203.25	202.73	206.72	201.38	200.92	215.25

#### 3.3.2 Future distribution projections

**Figure 6:**
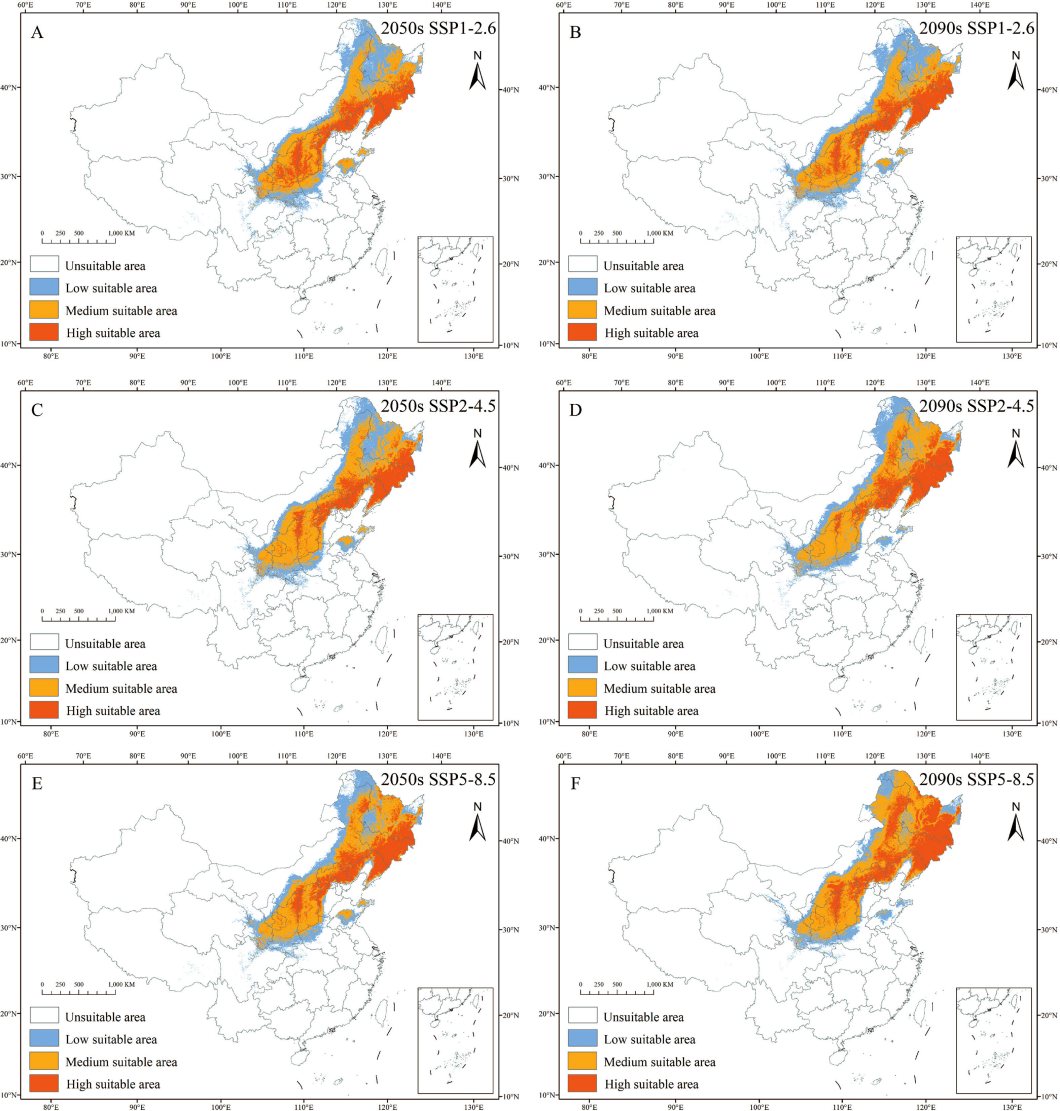
Distribution of suitable areas for *C. heterophylla* in China in ssp126 **(A, B)**, ssp245 **(C, D)**, ssp585 **(E, F)** scenarios in the 2050s **(A, C, E)** and 2090s **(B, D, F)**.

Projections indicate that under all scenarios, areas with low suitability will continue to shrink, while areas with moderate and high suitability will expand (**Figure 6**; **Table 4**). By the 2050s: Under SSP1-2.6, low suitability areas decrease by 40.38×10^4^ km², high suitability areas increase by 12.99×10^4^ km², and medium suitability areas increase by 19.79×10^4^ km²; Under SSP2-4.5, low-suitability areas decrease by 44.23×10^4^ km², medium-suitability areas expand by 26.2×10^4^ km², and high-suitability areas expand by 9.91×10^4^ km²; The SSP5-8.5 scenario shows a decrease of 46.95×10^4^ km² in low suitability areas, an increase of 24.74×10^4^ km² in moderate suitability areas, and an expansion of 18.08×10^4^ km² in high suitability areas. By the 2090s, SSP1-2.6 shows a 38.55×10^4^ km² reduction in low suitability zones, a 17.1×10^4^ km² expansion in moderate suitability zones, and an 11.98×10^4^ km² expansion in high suitability zones; SSP2-4.5 shows a 48.06×10^4^ km² loss in low-intensity areas, a 24.54×10^4^ km² expansion in moderate-intensity areas, and a 13.59×10^4^ km² gain in high-intensity areas; SSP5-8.5 shows a low-level reduction of 63.66×10^4^ km², but is accompanied by a significant expansion of 32.9×10^4^ km² in the moderately suitable zone and 35.16×10^4^ km² in the highly suitable zone. Among these scenarios, SSP5-8.5 exhibits the highest sensitivity to climate change due to its substantial increase in moderately and highly suitable habitats.

### 3.4 Changes in spatial patterns of *C. heterophylla* under different climatic scenarios

**Figure 7:**
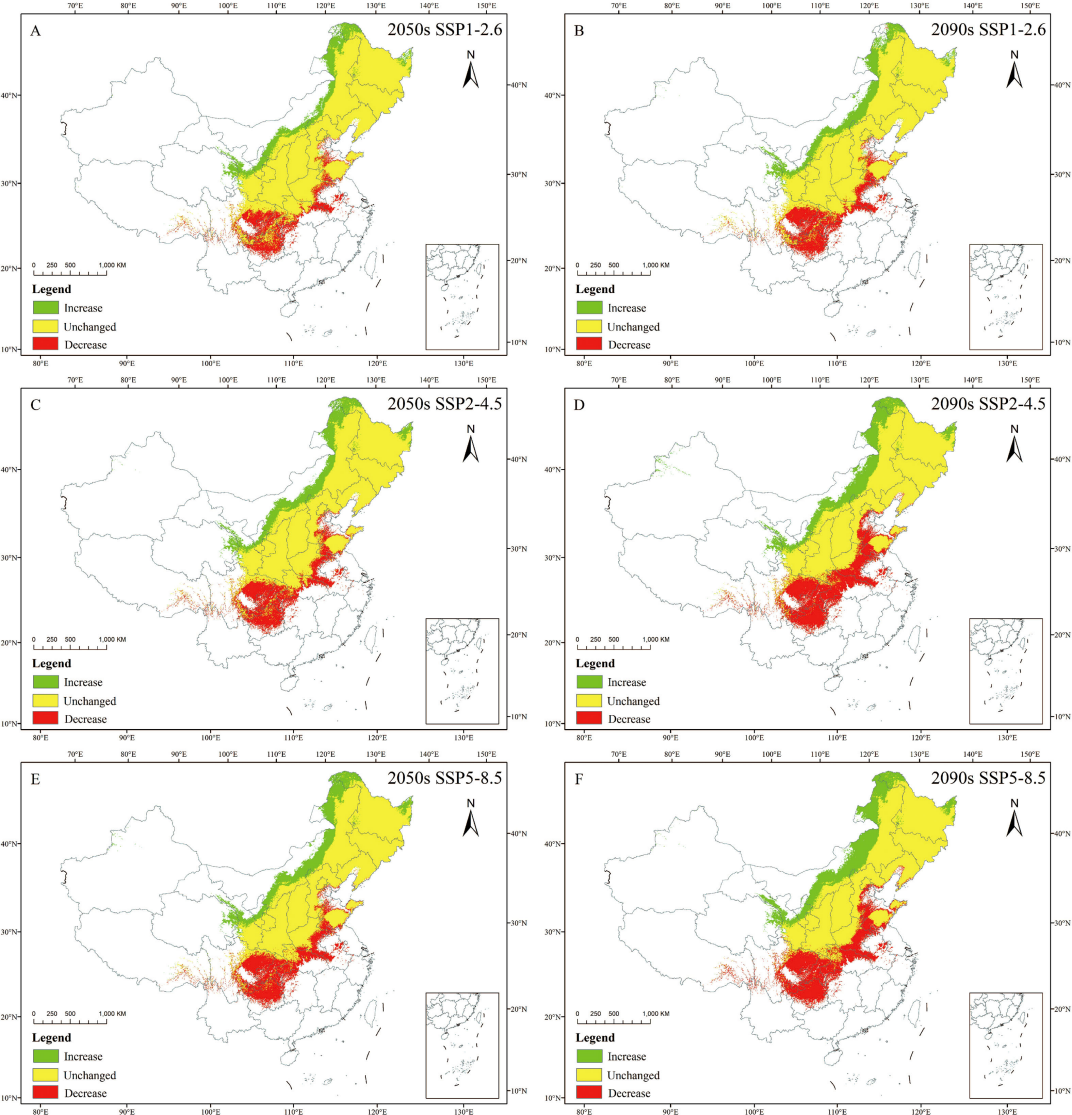
Spatial changes in *C. heterophylla* in China in ssp126 **(A, B)**, ssp245 **(C, D)**, ssp585 **(E, F)** scenarios in the 2050s **(A, C, E)** and 2090s **(B,D, F)**.

Future habitat suitability for *C. heterophylla* shows substantial overlap (69.20%~77.56% retention) with 1970–2000 suitable areas across all scenarios, mainly concentrated in Heilongjiang, Jilin, Liaoning, Beijing, Tianjin, Hebei, Shanxi, Shandong. Over all, the rate of habitat expansion ranges from 18.83% to 37.78%, with newly suitable areas mainly emerging in northern and high-altitude marginal regions. Conversely, range contraction occurs predominantly along the southern margins of the historical distribution. The highest increase is observed under the 2090s SSP5-8.5 scenario, while the smallest increase is observed under the 2050s SSP1-2.6 scenario. Suitable areas are expected to expand mainly in the northern regions of Heilongjiang, Inner Mongolia, northern Qinghai, Gansu, and northern Ningxia. The rate of loss is 22.44% ~ 30.70%, with the highest loss under the 2090s SSP2-4.5 scenario, and the lowest loss under the 2050s SSP1-2.6 scenario. The suitable area for *C. heterophylla* is anticipated to contract mainly in southern Hubei, southern Hunan, southern Henan, eastern Sichuan, and southern Guizhou (**Table 5**; **Figure 7**).

**Table 5:** Changes in habitat area of *C. heterophylla* across future climate scenarios.

Scenarios	Area/10^4^ km^2^			Change/%		
	Increase	Unchanged	Decrease	Increase	Unchanged	Decrease
2050s SSP1-2.6	39.71	163.54	47.32	18.83	77.56	22.44
2050s SSP2-4.5	45.62	157.11	53.75	21.64	74.51	25.49
2050s SSP5-8.5	52.71	154.01	56.85	24.99	73.04	26.96
2090s SSP1-2.6	42.05	159.33	51.54	19.94	75.57	24.44
2090s SSP2-4.5	55.01	145.91	64.95	26.09	69.20	30.80
2090s SSP5-8.5	69.12	146.13	64.73	37.78	69.31	30.70

Northern Heilongjiang, Inner Mongolia, northern Qinghai, Gansu, northern Ningxia, southern Hubei, southern Hunan, southern Henan, eastern Sichuan, and southern Guizhou represent core change zones for future habitat suitability shifts in China.

### 3.5 Centroid migration trends of *C. heterophylla* under different climatic scenarios

*C. heterophylla* distribution centroids exhibited consistent northward shifts across all scenarios. During the 1970–2000 period, the centroid was located near Dazhangjia Village, Longyao County, Xingtai City, Hebei Province (114.849°E, 39.409°N). Under the SSP1-2.6 scenario, the centroid is projected to shift to the vicinity of Zhoukoudian, Fangshan District, Beijing (115.94°E, 39.66°N) by the 2050s, and further toward Fengtai District, Beijing (116.402°E, 39.857°N) by the 2090s. Under the SSP2-4.5 scenario, it is expected to migrate to the area around Miyun District, Beijing (116.911°E, 40.598°N) by the 2050s, and eventually reach Huangtukang Township, Weichang Manchu and Mongolian Autonomous County, Chengde City, Hebei Province (117.697°E, 41.764°N) by the 2090s. Under the SSP5-8.5 scenario, the centroid will move to Datou Mountain Township, Weichang Manchu and Mongolian Autonomous County (117.269°E, 41.858°N) by the 2050s, and finally arrive at Tianqiao Town, Fengning Manchu Autonomous County, Chengde City (117.185°E, 41.014°N) by the 2090s.

## 4 Discussion

### 4.1 Key environmental determinants of habitat suitability of *C. heterophylla*

The results from the environmental variables replacement contribution analysis and jackknife test indicate that Bio16 (Precipitation of wettest quarter, 39.5%), Bio9 (Mean temperature of driest quarter, 22.2%), Alt (Altitude, 16.2%), and Bio3 (Isothermality, 7.1%) represent primary environmental factors governing the distribution of *C. heterophylla* (**Table 3**; **Figure 3**). However, discrepancies exist regarding the identification of primary environmental factors across different studies. Liu and Chen (2024) reported that the Bio6 (Minimum temperature of coldest month) and human footprint were the main drivers for hazelnut distribution. This divergence may arise from their analysis of multiple *C. heterophylla* species collectively rather than species-specific responses. The ecological adaptability of different *Corylus* species varies significantly, and pooled analyses may fail to capture their species-specific environmental requirements. Similarly, He et al. (2023) identified Bio4 (Temperature seasonality), Bio16 (Precipitation of wettest quarter), and Bio15 (Precipitation seasonality) as dominant factors, potentially because their study did not account for topographic influences. Topographic parameters, including altitude, slope, and aspect, significantly influence plant distribution patterns, particularly in regions with complex geomorphology, by mediating habitat suitability through their effects on localized climatic conditions and edaphic properties (Huang et al., 2024). Consequently, the integration of multidimensional environmental variables is critical for robust identification of the mechanistic drivers governing phytogeographic distributions.

Bio16 showed an optimal range of 300–450 mm, with the highest probability of occurrence peaking at 350 mm (**Figure 4**), reflects the species’ sensitivity to water supply during the critical summer fruit development stage. In many temperate regions, peak precipitation coincides with summer (Fick and Hijmans, 2017), providing essential soil moisture for nut filling and oil synthesis. Moderate rainfall ensures high yields and quality, while excessive rainfall leading to waterlogging can cause root asphyxiation and increase disease incidence (Marsal et al., 1997). Bio9 exhibited a suitable range of -12°C to 2°C, indicating strong adaptation to cold and dry winters. This physiological trait enables *C. heterophylla* to survive extreme winter temperatures as low as -30°C to -40°C (Erdogan and Mehlenbacher, 2000). Regarding Alt (Altitude), this study identified a constrained distribution range of 100–700 m, peaking at 300 m. Wang et al. (2020) reported a broader suitable altitude range of 0–3000 m for *C. mandshurica.* This divergence suggests a clear altitudinal niche partitioning between the two species, which may reduce direct competition and facilitate their coexistence in sympatric regions. Huo et al. (2016) demonstrated that the northern populations of *C. heterophylla* and *C. mandshurica* exhibit high climatic similarity, though *C. mandshurica* generally occurs at higher elevations than *C. heterophylla* in sympatric regions. This finding is in line with the results of this study. Bio3 has an optimal range of 24 to 30. As the ratio of Bio2 (Mean diurnal temperature range) to Bio7 (Temperature annual range), this variable ecologically reflects the relative magnitude of diurnal versus seasonal temperature variation. The identified optimum indicates that the species thrives in regions with pronounced annual temperature seasonality. This climatic preference aligns with the physiological traits of the *C. heterophylla* as a temperate deciduous shrub, particularly its dependence on cold winters (Fadón et al., 2020). Sufficient low-temperature accumulation is crucial for meeting the “chilling requirement” necessary to break dormancy, thereby ensuring synchronized spring bud break and normal inflorescence development (González De Andrés et al., 2023). Conversely, in regions with Bio3 values significantly exceeding 30 (e.g., maritime climates), elevated winter temperatures may fail to meet cold requirements, triggering dormancy disorders that impair normal growth and development (Luedeling et al., 2011). Thus, the distribution and adaptation of *C. heterophylla* depend not only on growing-season temperature conditions but critically on temperature patterns exhibiting pronounced seasonal rhythms to synchronize its phenological processes with the external environment.

### 4.2 Potential impacts of climate change on suitable habitats for *C. heterophylla*

The analysis of environmental variables and their impact on the potential distribution of *C. heterophylla* under 1970–2000 and future climate scenarios provides significant insights into the species’ ecological adaptability and future prospects. Using the optimized MaxEnt model, we predict the species’ geographic distribution under different time periods. Under 1970–2000 climatic conditions, *C. heterophylla* is primarily distributed in Northeast China (Changbai Mountains and Xiao Xing’anling ranges), North China (Yanshan Mountains), and Northwest China (northern slopes of the Qinling Mountains) (**Figure 5**). The total suitable area covers 210.85×10^4^ km² (21.96% of China’s land area), with the highly suitable area accounting for 39.09×10^4^ km² and being concentrated in Jilin, Liaoning, Hebei, Shanxi provinces (**Table 4**). Future climate scenario simulations consistently project a contraction in the total suitable habitat range of *C. heterophylla*. This trend aligns with earlier studies reporting accelerated range loss in its southern low-elevation populations (Yang et al., 2022). Model results further suggest that although low-suitability areas will gradually decrease, medium- and high-suitability zones are expected to expand in a spatially heterogeneous manner across climate scenarios, particularly in regions such as northern Heilongjiang, northern Inner Mongolia, and northwestern provinces including Gansu and Ningxia (**Figure 7**). This latitudinal and altitudinal expansion is driven by combined effects of climate warming and projected shifts in precipitation patterns. Altered thermal and moisture conditions collectively enable previously cold-limited northern and high-elevation areas to gradually meet the eco-physiological requirements of *C. heterophylla* (Álvarez-Álvarez et al., 2025). These newly expanded areas represent potential future habitats for the *C. heterophylla*, holding critical conservation value for maintaining its population persistence and mitigating biodiversity loss caused by the species’ southern distribution decline.

### 4.3 Spatial change patterns and centroid shift analysis

Under future climate change scenarios, the suitable distribution range of *C. heterophylla* exhibits a bipolar migration pattern characterized by “northward expansion and southward contraction” (**Figure 7**). Newly suitable habitats are predominantly distributed in northern Heilongjiang, northern Inner Mongolia, Gansu, Qinghai, and other northwestern regions, whereas substantial habitat loss is observed in southern China, including Hubei, southern Henan, and Sichuan. Compared to the SSP1-2.6 and SSP2-4.5, SSP5-8.5 scenario shows the most significant changes in suitable areas. This can be attributed to the substantial increases in both precipitation and temperature across northern China under this high-emission pathway (Zhou et al., 2020), which collectively create thermal hygric conditions that more closely match the species’ requirements during critical growth phases (Fick and Hijmans, 2017). In contrast, under the SSP2-4.5 and SSP1-2.6 scenarios, the more moderate warming and limited precipitation increases are insufficient to generate equivalent habitat gains in the north. Consequently, the net change in these scenarios is dominated by habitat losses in the southern parts of the range, resulting in a greater overall reduction in total suitable area. CMIP6-based projections of future global temperatures reveal persistent warming trends across all emission scenarios, albeit with significant variations in magnitude. Zhao et al. (2021) further estimate a global temperature increase ranging from 0.6 to 7.8 °C, which may exacerbate the frequency and intensity of extreme climate events. Additionally, CMIP6 models project increased precipitation across most regions of China, with the most substantial enhancement occurring under the high-emission scenario (SSP5-8.5) (Álvarez-Álvarez et al., 2025). The northward migration of suitable habitats can be attributed to synergistic improvements in hydrothermal conditions in northern regions, which optimize the growth environment for *C. heterophylla.* Conversely, in the southern parts of the current distribution range, future projections indicate that precipitation may exceed the species identified optimal upper threshold of 450 mm (**Figure 4**; Xu et al., 2022). Such excessive rainfall could result in periodic soil waterlogging, elevating the risk of root hypoxia—a condition known to be detrimental to *C. heterophylla* (Bailey-Serres and Voesenek, 2008).

**Figure 8:**
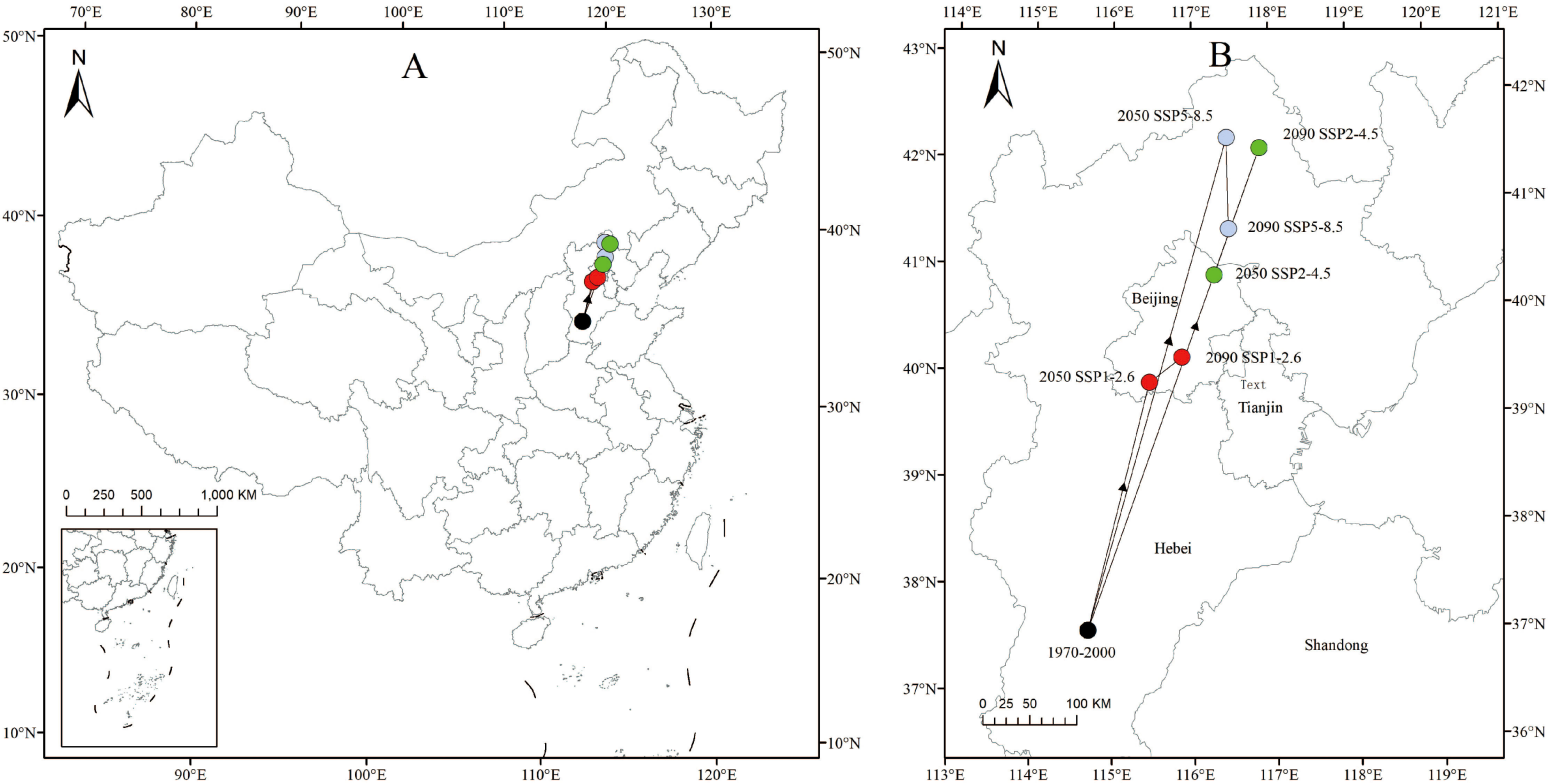
The geographical distribution change of the centroid of *C. heterophylla* suitable area under different climate scenarios [**(B)** is the expansion of part **(A)**].

The distribution centroid of *C. heterophylla* demonstrates a consistent northward migration trend under all future climate scenarios relative to its historical position (1970–2000) in Hebei Province (**Figure 8**). This migratory trend is most pronounced under the higher-emission scenarios (SSP2-4.5 and SSP5-8.5) and is strongly linked to projected changes in precipitation patterns. Climate models indicate a general increase in growing-season precipitation across northern China (Zhou et al., 2020). This enhanced water supply may transform previously water-limited areas in the north into new suitable habitats for *C. heterophylla*. Under high-emission scenarios, while traditional distribution areas like North China may experience intensified seasonal droughts and precipitation uncertainty, some more northern regions could benefit from modest precipitation increase or greater climatic stability (Zhang et al., 2021). This shift in precipitation patterns favors a contraction of suitable habitat toward these more favorable high-latitude zones. Conversely, in the southern parts of the current range, increased precipitation is projected to frequently exceed the species’ optimal upper threshold (450 mm; **Figure 4**), leading to waterlogging stress and habitat degradation. Supporting this, Wang et al. (2019) documented upward shifts in suitable ranges for most plant species in Changbai Mountain in response to rising temperatures and altered precipitation patterns, with significantly greater migration rates and distances under high-emission scenarios compared to low-emission ones. These findings collectively suggest that enhanced warming under high-emission scenarios likely drives more substantial displacements of *C. heterophylla*’s distribution center.

## 5 Conclusions

This study employed an optimized MaxEnt model to predict the potential distribution shifts of *C. heterophylla* under current and future climate change scenarios. Bio16 (Precipitation of wettest quarter), Bio9 (Mean temperature of driest quarter, 15.5%), Alt (Altitude, 15.3%), and Bio3 (Isothermality, 7.1%) were identified as core environmental factors constraining *C. heterophylla*’s distribution, revealing the species’ strong dependence on seasonal hydrothermal patterns. Although the total suitable habitat area is projected to decrease under future climates, the extent of moderately to highly suitable habitats is expected to increase, accompanied by a pronounced northward shift in the distribution centroid. These findings provide practical insights for the conservation and utilization of *C. heterophylla* germplasm resources. It is recommended to proactively establish climate-resilient conservation bases in northern expansion zones to ensure sustainable use and enhance production potential under future climates. In southern contraction zones, a “defensive conservation” strategy should be adopted, prioritizing the establishment of germplasm nurseries and continuous population monitoring to safeguard genetic diversity.

## Data Availability

The original contributions presented in the study are included in the article/**Supplementary Material**. Further inquiries can be directed to the corresponding authors.
