# Supplementary material for: Predicting the potential distribution of Corylus heterophylla in China under future climate change using an optimized MaxEnt model
Source: Front Plant Sci. 2025 Nov 17;16:1668828. doi: 10.3389/fpls.2025.1668828 (PMC12667186; doi:10.3389/fpls.2025.1668828)
Supplement: Supplementary file 1 [file Table1.docx]

| Variable code | Environmental factor | Unit |
| --- | --- | --- |
| Bio1 | Annual mean temperature | ℃ |
| Bio2 | Mean diurnal temperature range | ℃ |
| Bio3 | Isothermality (BIO2/BIO7) (×100) | - |
| Bio4 | Temperature seasonality (standard deviation ×100) | - |
| Bio5 | Maximum temperature of warmest month | ℃ |
| Bio5 | Minimum temperature of coldest month | ℃ |
| Bio7 | Temperature annual range | ℃ |
| Bio8 | Mean temperature of wettest quarter | ℃ |
| Bio9 | Mean temperature of driest quarter | ℃ |
| Bio10 | Mean temperature of warmest quarter | ℃ |
| Bio11 | Mean temperature of coldest quarter | ℃ |
| Bio12 | Annual precipitation | mm |
| Bio13 | Precipitation of wettest month | mm |
| Bio14 | Precipitation of driest month | mm |
| Bio15 | Precipitation seasonality | mm |
| Bio16 | Precipitation of wettest quarter | mm |
| Bio17 | Precipitation of driest quarter | mm |
| Bio18 | Precipitation of warmest quarter | mm |
| Bio19 | Precipitation of coldest quarter | mm |
| Alt | Altitude | m |
| Slo | Slope | % |
| Asp | Aspect | ° |
| T_bs | **Base Saturation (Topsoil)** | % |
| T_silt | Silt content in topsoil (0–30 cm) | % |
| T_sand | Sand content in topsoil | % |
| T_ph | pH in water solution (topsoil) |  |
| T_oc | Organic carbon content (topsoil) | % |
| T_CEC_soil | Cation Exchange Capacity (topsoil) | cmol(+)/kg |
| T_clay | Clay content in topsoil | % |
| Awc_Cless | Available water capacity | % |
| REF_Depth | Reference depth | cm |
